# Acceptability of a standalone written leaflet for the National Health Service for England Targeted Lung Health Check Programme: A concurrent, think‐aloud study

**DOI:** 10.1111/hex.13520

**Published:** 2022-06-02

**Authors:** Mbasan Jallow, Georgia Black, Sandra van Os, David R. Baldwin, Kate E. Brain, Michael Donnelly, Samuel M. Janes, Clara Kurtidu, Grace McCutchan, Kathryn A. Robb, Mamta Ruparel, Samantha L. Quaife

**Affiliations:** ^1^ Research Department of Behavioural Science and Health University College London London UK; ^2^ Department of Applied Health Research University College London London UK; ^3^ Department of Respiratory Medicine Nottingham University Hospitals NHS Trust, City Hospital Nottingham UK; ^4^ Division of Population Medicine Cardiff University Cardiff UK; ^5^ Centre for Public Health Queen's University Belfast Belfast UK; ^6^ Lungs for Living Research Centre, UCL Respiratory, Division of Medicine University College London London UK; ^7^ Institute of Health and Wellbeing University of Glasgow Glasgow UK; ^8^ Wolfson Institute of Population Health, Barts and The London School of Medicine and Dentistry Queen Mary University of London London UK

**Keywords:** decision aid, decision‐making, informed choice, lung cancer screening

## Abstract

**Background:**

Many countries are introducing low‐dose computed tomography screening programmes for people at high risk of lung cancer. Effective communication strategies that convey risks and benefits, including unfamiliar concepts and outcome probabilities based on population risk, are critical to achieving informed choice and mitigating inequalities in uptake.

**Methods:**

This study investigated the acceptability of an aspect of NHS England's communication strategy in the form of a leaflet that was used to invite and inform eligible adults about the Targeted Lung Health Check (TLHC) programme. Acceptability was assessed in terms of how individuals engaged with, comprehended and responded to the leaflet. Semi‐structured, ‘think aloud’ interviews were conducted remotely with 40 UK screening‐naïve current and former smokers (aged 55–73). The verbatim transcripts were analysed thematically using a coding framework based on the Dual Process Theory of cognition.

**Results:**

The leaflet helped participants understand the principles and procedures of screening and fostered cautiously favourable intentions. Three themes captured the main results of the data analysis: (1) Response—participants experienced anxiety about screening results and further investigations, but the involvement of specialist healthcare professionals was reassuring; (2) Engagement—participants were rapidly drawn to information about lung cancer prevalence, and benefits of screening, but deliberated slowly about early diagnosis, risks of screening and less familiar symptoms of lung cancer; (3) Comprehension—participants understood the main principles of the TLHC programme, but some were confused by its rationale and eligibility criteria. Radiation risks, abnormal screening results and numerical probabilities of screening outcomes were hard to understand.

**Conclusion:**

The TLHC information leaflet appeared to be acceptable to the target population. There is scope to improve aspects of comprehension and engagement in ways that would support informed choice as a distributed process in lung cancer screening.

**Patient or Public Contribution:**

The insight and perspectives of patient representatives directly informed and improved the design and conduct of this study.

## 1 INTRODUCTION

Lung cancer leads cancer mortality worldwide^1^ and disproportionately so within lower socioeconomic communities.^2^ Early detection, using low‐dose computed tomography (LDCT) screening, significantly reduces lung cancer mortality among high‐risk adults.^3^, ^4^ For example, in the National Lung Screening Trial there were 20% fewer deaths from lung cancer among high‐risk adults within the screening arm (screened annually with LDCT) than the control arm (screened annually with chest X‐ray).^3^ Consequently, several countries are implementing national screening programmes for those above a certain threshold of risk for lung cancer, which is typically defined as being aged 55–80 years with a significant and relatively recent smoking history, although other individual risk factors are also taken into consideration by some programmes, including medical and family history, some demographics and other exposures. In the United Kingdom, the National Health Service for England (NHSE) launched a ‘Targeted Lung Health Check (TLHC) Programme’, offering LDCT screening to adults aged 55–74 years at increased risk of lung cancer in selected areas of England ahead of the UK National Screening Committee's decision.

To ensure that high‐risk groups make an informed choice about whether to attend, screening programmes must design effective public‐facing communication strategies and information materials. Informed choice is defined as a decision made with adequate knowledge, which is consistent with the decision‐maker's values and ultimately enacted.^5^ While central to UK health policy,^6^ informed choices are challenging to achieve through advanced written communication strategies. Screening‐eligible individuals need to understand complex risks and benefits of screening, including unfamiliar concepts like overdiagnosis,^7^ with outcome probabilities based on population rather than individual risk. People who find this information difficult to understand may particularly struggle if they experience fearful emotional responses to screening,^8^, ^9^ and have lower literacy or numeracy.^10^ Together, these factors increase the likelihood that people will misinterpret, avoid or disregard information materials due to emotional and cognitive influences on information processing and attention. Indeed, a systematic review^11^ found that while several US‐based studies of decision support tools for lung cancer screening increased overall knowledge scores, key elements of lung screening knowledge remained misunderstood. These included the frequency of false‐positive results and the size of the lung cancer mortality benefit. In one study,^12^ subjectively rated knowledge of the risks and benefits of screening (i.e., participants' perception of their knowledge) was higher than their objectively measured knowledge.

Dual Process Theories of cognition provide a useful framework for exploring how individuals interpret and understand written cancer screening information. They distinguish conceptually between two interacting cognitive systems.^13^, ^14^ System 1 concerns fast, automatic, and intuitive thinking based on heuristics such as emotional responses, stereotypes, experiences and assumptions. System 2 concerns slower, analytical and effortful thinking, which can override the impulses of System 1, which is needed to achieve an informed decision. Evidence suggests that high information burden and leaflet styles that require System 2 ‘deliberative thinking’ can serve to disengage people in lower socioeconomic groups^15^ or lead individuals to be guided by their System 1 emotions or pre‐existing assumptions.^10^ In the Lung Screen Uptake Trial, advanced provision of detailed written information did not improve screening knowledge at the appointment compared with a low burden information leaflet.^12^ Furthermore, in the colorectal cancer screening context, 20% of attendees and 63% of nonattendees reported that they did not read the invitation leaflet,^16^ suggesting that informed choice may be the lowest among nonparticipants.

In line with the Medical Research Council's Framework for developing complex interventions,^17^ this study aimed to understand the acceptability of using a standalone written information leaflet to invite and inform high‐risk adults about lung cancer screening. Acceptability was explored in terms of how a diverse sample of high‐risk adults responded to, engaged with and comprehended NHSE's TLHC leaflet using the Dual Process Theory of cognition.

## 2 METHODS

### 2.1 Study design

This was a qualitative study. Semi‐structured interviews used a concurrent think‐aloud method to observe participants' responses, comprehension and attentional engagement while reading NHSE's TLHC leaflet (File S1), as well as the underlying emotional and cognitive processes.^18^

This study was carried out between November 2020 and January 2021, when NHSE's TLHC programme had begun operating at 23 sites in England, but there was no NHS‐provided lung cancer screening in Northern Ireland, Scotland or Wales. During this period of time, there was a global COVID‐19 pandemic and the UK government imposed two national lockdowns (5th November and 6th January) to restrict nonessential in‐person activity to reduce the spread of the virus.

### 2.2 Participants

The eligibility criteria were: (i) current or former daily smokers (quit ≤15 years), (ii) aged 55–75, (iii) never participated in lung cancer screening and (iv) resident in the United Kingdom. Data saturation was achieved with a sample of 40 participants.

### 2.3 Sampling

In line with norms for qualitative research,^19^ we recruited a sample of 40 participants, purposively selected for diversity in terms of age, gender, smoking status, education level (as an individual marker of socioeconomic position) and ethnicity. Quotas were set to recruit 10 participants from each of the four UK nations and then within each individual nation, to ensure an even split by gender and smoking status, at least two‐thirds with the lowest level of educational attainment (finished school, aged ≤16), a range of ages, and at least 30% of black, Asian, mixed or other ethnic backgrounds. A specialist recruitment company (Taylor McKenzie Ltd.) recruited participants directly from their in‐house database of over 12,000 individuals.

### 2.4 Ethical considerations

University College London's Research Ethics Committee granted approval (reference: 17701/001).

### 2.5 Procedure

Our research team included health psychologists, behavioural scientists, clinicians and decision‐makers involved in the TLHC programme. Interviews were conducted by telephone or video call by M. J. Participants were sent an information sheet, consent form and three sealed envelopes (to be opened during the interview) containing: (i) practice leaflet, (ii) NHSE TLHC (lung cancer screening) leaflet developed by NHS England (File S1) and (iii) extracts from alternative lung screening resources. Participants provided consent verbally and were reimbursed for their time and expenses by the specialist recruitment company (£40). Interviews were audio‐recorded and transcribed verbatim.

The interviews comprised four stages (see File S2 for interview schedule):

1. *Warm‐up questions* included usual sources of information, role in decision‐making and health information preferences.
2. *Practice of the ‘Think‐Aloud’ approach* used an unrelated leaflet on car rentals. Positive reinforcement (i.e., assuring participants when verbalizing their thoughts and feelings that this was exactly what was required) and the use of prompts (i.e., to remind participants to verbalize their thoughts and feelings) encouraged and familiarized participants with verbalizing thoughts and feelings.
3. *‘Think‐Aloud’ task* required participants to read the TLHC leaflet and verbalize their thoughts, feelings, interpretation and comprehension. Participants were asked to imagine that they had just received the leaflet in the mail alongside an invitation to take part in the NHS England TLHC programme. The researcher passively observed, sometimes using prompts to ensure participants covered priority aspects of the leaflet while retaining participant‐led exploration.^18^
4. *Follow‐up probes* were used to elaborate on participants' understanding and responses. Extracts from alternative lung cancer screening information resources were shown to participants to explore their preferences for alternative styles and methods of presenting similar information to that shown in the TLHC leaflet.

### 2.6 Data analysis

A combination of inductive and deductive approaches was used to analyse the data. First, a skeletal coding framework was created based on Systems 1 and 2 of the Dual Process Theory of cognition.^13^ After familiarization with the transcripts, three were inductively coded independently by M. J. and S. L. Q. in NVivo 12. Both M. J. and S. L. Q. are experienced, qualitative researchers. S. L. Q. has experience using Dual Process Theory in research settings. These inductive codes were categorized within the skeletal framework, with some overlapping different categories, and compared through discussion. After minor revisions to the framework, M. J. inductively coded the remaining transcripts. Table 1 provides definitions for the engage, respond and comprehend categories used to organize inductive codes.

**Table 1:** Descriptions of categories for organizing inductive codes

Categories for organizing inductive codes	Description
Response	We considered responses in terms of emotion, interpretation and anticipated behaviour. In the context of informed choice, an effective leaflet should minimize adverse emotional reactions that reduce information receptivity and comprehension.
Engagement	We considered how participants approached the leaflet, and what types of information and sustained attention versus types that were overlooked. Sustained attention is crucial to the success of the leaflet in supporting informed choice.
Comprehension	We considered how well different aspects of the information were understood, any assumptions or areas of misunderstanding, confusion or conflation and the effort involved in understanding the information. We also examined how participants interpreted the information to understand the causes of, and solutions for, misunderstandings.

Following this, each inductive code was sorted during a virtual group exercise (M. J., S. L. Q., G. B., S. V. O.) into columns relating specifically to the research aims (i.e., how people engage with, respond to and comprehend the information) regardless of their position within System 1 and System 2. This included an in‐depth discussion of the respective quotes as the basis for each code and theme. Additional columns included recommendations and preferences for information provision.

### 2.7 Reflexive account

It is important to reflect upon the ways in which the characteristics of the research team and research context could have unintentionally introduced bias into the research process. The researcher who carried out the interviews was younger than the participants, had no smoking history (although this was not disclosed to participants), and was approaching participants as a university‐based researcher. It is possible that participants may have been less willing to verbalize their thoughts and feelings and were less open to disclosing difficulties with comprehension due to these differences and the interviewer's position as a university academic. These were not naturally occurring conversations and were being recorded for research purposes, which may also have adversely affected participants' openness.

## 3 RESULTS

### 3.1 Sample characteristics

Forty participants (mean age: 60.5 years, range: 55–73; see Table 2) were interviewed, 10 from each UK nation (England; Wales, Northern Ireland, Scotland). Their characteristics varied: 50% were women, 62.5% were of white ethnicity, 20% were of black ethnicity and 10% South Asian. Most participants (67.5%) finished school, aged ≤16 years. Current and former smokers were evenly represented with the time since quitting, ranging from 10 months to 15 years.

**Table 2:** Sample characteristics (*N* = 40)

Characteristic	*n* (%)
Gender	
Male	20 (50.0)
Female	20 (50.0)
Age	
55–59	20 (50.0)
60–64	12 (30.0)
70–73	8 (20.0)
Ethnicity	
White (British/Irish/Scottish/other)	25 (62.5)
Black (British/African/Caribbean)	8 (20.0)
Asian (British/Pakistani/Indian)	4 (10.0)
Mixed (Black Caribbean and White British)	2 (5.0)
Egyptian	1 (2.5)
Educational attainment	
Finished school, aged ≤16 years	27 (67.5)
Completed O or A levels	8 (20.0)
Further education	4 (10.0)
Bachelor's degree	1 (2.5)
Smoking status	
Current smoker	20 (50.0)
Former smoker (10 months–2 years)	3 (7.5)
Former smoker (3–5 years)	5 (12.5)
Former smoker (10–15 years)	12 (30.0)

### 3.2 Thematic overview

Themes were organized under three categories (1) responses to the leaflet contents, (2) engagement with the leaflet and (3) comprehension of the images and written information, and their description is supported by longer illustrative quotes shown in Tables 3, 4, 5. Participant codes (e.g., M_65_CS) represent participants' gender (M = male, F = female), age and smoking status (CS = current smoker, FS = former smoker).

**Table 3:** Quotes illustrative of response themes

Themes	Illustrative quotes
1. Weighing fears of screening with the benefits of early diagnosis	**F_62_CS**: ‘It's a bit scary when it says the offer of the lung cancer screening scan, but then I suppose that's a good thing because… if they do think there's a problem it could be caught early enough’. **M_71_FS**: ‘I've lost three brothers and they was all like suddenly, from finding out what was wrong with them to actually dying was only a matter of months. but of the three of them, they was terrified of going to the doctors you see and this is what I used to tell them, if you would have gone to the doctors something could've been done. But you left it that late’.
2. Reassurance about a comprehensive process managed by specialists	**F_55_CS**: ‘I like this page, because they give the outline, it's clear what's going to happen from coming in to leaving, and they're also telling you how long it's going to take. Also, it's quite nice that they're saying you can bring family or a friend or partner with you, because some people do get nervous, and it's nice to have somebody’. **M_60_CS**: ‘the fact it says nurse is strangely enough, that I'd be more liable than if it said GP for some reason, because it seems less formal, maybe it's psychological, the fact it's a nurse, there's a trust there’. **M_70_FS**: ‘it goes back to your GP, doesn't it? Because it should start with your doctor and goes back there…everything goes back to the GP, that's because he's the first one you go to’.
3. Anticipatory anxiety about screening results and further investigations	**M_61_CS**: ‘Well they could be frightening, you could get good news, you could get news of a further scan needed, which would be a worry until you would have that over. If you got results with abnormal result, that would be a worry for a while, or even the incidental finding, there could be something else’. **F_61_CS**: ‘I think that's just a bit scary… I would prefer to have either the nurse talk to me about that or a doctor talk to me about that, rather than it be written down like this’. **F_55_CS**: ‘I'm not sure I want this. Because… you have to wait four weeks for your results, and then if there's shadows on your lungs, which it's saying it's probably something harmless but it could be more serious, you, you've then got to wait another three months… And then you've got another four weeks to wait for your results again. That's five months…. It would freak you out waiting for the results…. And then they tell you this, they've found shadows for this, abnormal, but don't worry love, we'll get you back in again in three months' time to have another one. And then in three months' time they tell you, we'll give you your results in four weeks’.
4. Images intended to support comprehension provoked negative emotions	**F_59_FS**: ‘So if you've got lung cancer does your lungs go from that to that like a flower? It must be, so it looks as though they change shape, swell up, or it, … have they cut your lung in half so that you can see what it's like inside?’ **M_54_FS**: ‘I've ignored the picture, because that doesn't mean anything whatsoever. So the first thing I'd do,… I'd ignore the picture, and go, turn the page’. **F_55_CS**: ‘you have a picture [of CT scanner] in your head, and it's totally different from what you're seeing’.
5. Fatigue with smoking information	**M_57_CS**: ‘And then it's the old issue of what can I do to reduce my risk and I know what it is, smoking’. **M_60_CS**: ‘I wouldn't log on to one of these websites…I wouldn't dial 0300, because I've dialled it before and I've tried. And I've tried the patches and I've tried the vapes. I've tried all these things… I personally just can't stop regardless of how many websites I log into or, the Smokefree helplines I ring… this particular addiction, is just a bit too strong really’.

*Note*: Participant codes (e.g., M_65_CS) represent participants' gender (M = male, F = female), age and smoking status (CS = current smoker, FS = former smoker).

**Table 4:** Quotes illustrative of engagement themes

Themes	Illustrative quotes
1. Enough information to engage attention, support autonomous consideration and initiate shared decision‐making	**F_55_CS**: ‘It's in my hands, my decision is up to me, but they're just helping you, to give you the facts, the information to make that decision, but it's still your decision…and that's what I like about the top, how it's saying, helping you make a decision. It's not forcing you to do anything’. **F_56_CS**: ‘it'd make me want to go forward with it….I think I'd read this and I'd go for the scan. But as far as following anything up with me further, I think this initiates you to go for the scan… I think any further follow up, you would follow up from this, maybe onto the internet’. **M_57_CS**: ‘the beauty of this is I can take this with me but I can also give this to my nearest and dearest and say this is why I'm doing, this is why I'm going and it's clear enough for everyone to be reading the same information’.
2. Attentional bias towards incidence and early detection messages	**M_71_CS**: ‘That's quite impressive actually, isn't it? Yeah. At least one more person for every 250 people will survive lung cancer if they had not been screened… that's good isn't it? That's quite impressive’. **F_63_FS**: ‘Well I didn't know that lung cancer was the most common types of cancer. That's really shocked me’. **M_71_CS**: ‘I mean everybody knows, if you find it early you've got a bigger chance, everybody knows that, so. I personally would take one anyway, because everybody knows the earlier the better’.
3. Known risks downplayed, but unfamiliar harms prompted deliberative thinking and concern about the screening reliability	**F_56_CS**: ‘Yeah because we all know that there's radiation in anything you're doing and that isn't there, …. they won't perform it if it was, like I said if it outweighed the odds of it being no good for you’. **F_62_FS**: ‘So if there's no cancer found then why do they done the operation? So that's no good because they make sure, they have to make sure if by the biopsy and that's false cancer, false operation. It's not right’. **M_56_CS**: ‘they can't even get the testing right, what's the point? And I'm not been given cancer drugs and cancer treatment for something that I haven't even got. I'm not having my life disrupted for something that I haven't even got… you're taking a 13% chance of that happening or whatever, you know? Because, that's what would put me off’. **F_58_FS**: ‘if you're going to be overdiagnosed and put you through a worrying time, thinking you've got lung cancer that's not going to cause you harm. If you're going to be so worried, how high, what rate does that happen at?’.
4. Engagement in symptom appraisal and awareness	**F_70_CS**: ‘short of breath. No I haven't got any of that. Coughing or change in your normal cough, coughing blood, no haven't got all of these, short of breath, no I haven't lost weight, no and I'm still eating, putting on bloody weight’. **F_59_FS**: ‘persistent cough, yes, coughing up blood, I've heard of that, tiredness or weight loss. Oh, so it can cause weight loss, is that because your throat hurts and you can't eat, or? I don't know. An ache or pain when breathing or coughing, yes, anything to do with my throat I'd be worried, appetite loss, yes. I wouldn't of put appetite loss to lung cancer’. **M_56_CS**: ‘I'm surprised they're saying there's usually no signs or symptoms, because usually if there's something up with the body you'll find something that will alert you to it’.

*Note*: Participant codes (e.g., M_65_CS) represent participants' gender (M = male, F = female), age and smoking status (CS = current smoker, FS = former smoker).

**Table 5:** Quotes illustrative of comprehension themes

Themes	Illustrative quotes
1. Understood the principle of an LHC and lung cancer screening	**F_70_FS**: ‘I like the way they do one, two, three, four….I like the headings and there's spaces in between and it's not too technically worded, I can understand everything that they're saying, it's quite explicit’. **F_55_FS**: ‘is this going to be a routine something in the way, when you're over as certain age, you get, you have your bowels tested regular after a certain age, is a lung screening thing going to become like that’. **M_57_FS**: ‘it tells you everything that's' going to be, what's going to be used and how it's done. And again, if you get in at an early stage you may get the cancer cells which is good as well’.
2. Understood the types of LHC results but assumed to clinically indicate the screening	**F_58_FS**: ‘Right, so you may or may not be offered a lung screening, cancer screening scan. So I don't know, I'm a wee bit confused, are the, why they only offer it to certain people… should it not just be beneficial to have it anyway?’ **F_56_CS**: ‘I think that should be put in, there's nothing available unless you've got a pronounced problem with your doctor, they're not going to just send you for a lung health check’. **M‐61_CS**: ‘Three options, no problems found, maybe a slight problem, they'll refer you to your GP, or if there's something there, they'll offer you a lung cancer screening scan, which I think can only be good’.
3. Misunderstood false negatives to be interval cancers	**F_59_FS**: ‘it still doesn't tell you why it could be missed because it said it can start growing after screening, well, that doesn't mean that they're missing it does it, that means it's not there when you have the scan’. **F_55_CS**: ‘Sounds like you can get cancer tomorrow, after screening, because you've had the radiation and it's made you get cancer’.
4. Poor understanding of radiation exposure due to an unfamiliar comparison	**M_54_FS**: ‘a CT scan's about the same as about one year's worth of radiation in the natural environment. I wouldn't have a clue what that would mean. But if it was compared to the amount, that maybe a CT scan is equivalent to ten X‐rays, I'd probably understand that better’. **M_73_CS: **‘Well, what harm is in having a screening. As I say it's only like a low X‐ray, so you don't worry about having an Xray when you break your leg, do you, so. So I don't think, I can't see any harm in having it’.
5. Conflating understanding of the different types of abnormal lung cancer screening results	**F_55_CS**: ‘Isn't a further scan needed [the same as] an abnormal result? I don't know… they've seen something abnormal and you're going to need further tests. But on the further scan needed, it could be more serious, so what's the difference with what they're finding?’. **M_58_FS**: ‘incidental findings means there's something there but it's not going to be serious’. **F_57_CS**: ‘I like the bit that it can, picks up something even though they do not have lung cancer, obviously a false positive which means you've got something else wrong with your lungs and they can look at a further test’.
6. Outcome probabilities engaged deliberative thinking but overwhelmed those who found them too complex	**F_59_FS**: ‘I think it's easy to look at [icon array], you've got your thing there with all the colours’. **F_55_FS**: ‘So are they trying to tell me that lung cancer is not that high in the population or?… the way I'm reading it it's saying to me that three quarters of the population won't result in lung cancer but the other quarter will’. **M_57_CS**: ‘this really takes some looking at now and thinking about, lung cancer if they've not been screened…. Wow, this is a lot of information for my brain so now I feel some form of obsessive compulsive need to work out the percentages’.

*Note*: Participant codes (e.g., M_65_CS) represent participants' gender (M = male, F = female), age and smoking status (CS = current smoker, FS = former smoker).

Abbreviation: LHC, Lung Health Check.

### 3.3 Responses to the leaflet's contents

The dominant responses to the leaflet were System 1 type emotions and assumptions, with fears about abnormal screening results and further investigations weighed against the reassuring role of specialist healthcare professionals (see Table 3). Particular types of images exacerbated those fears, and messages about smoking cessation provoked fatigue. However, the relative benefit of early detection prompted System 2 type reflective thinking, which in some cases overcame initial emotional reactions.

**1. Weighing fears of screening with the benefits of earlier diagnosis**

3.4

The description of the Lung Health Check (LHC) and lung cancer screening scan initially evoked fear among some participants who described the prospect as ‘frightening’ and ‘depressing’. However, reflecting upon the information about the benefits of early detection led most to perceive the LHC as a ‘very good’ thing. The explanation of early detection reducing the risk of dying, as well as recalled experiences of early and late diagnosis among family and friends, motivated some to attend.

**2. Reassurance about a comprehensive process managed by trusted specialists**

3.5

There was a broad sense of reassurance regarding the LHC and LDCT screening procedure, stemming from the clarity of descriptions and reassuring details (e.g., bringing friends/family, painless nature of scan) that set clear expectations of a straightforward process. Although many had not experienced a computed tomography (CT) scan, some likened the process to getting an X‐ray, similar health checks and other cancer screening programmes, supporting familiarity and trust. The approach to offering LDCT lung cancer screening within an LHC and the potential for screening to identify other conditions were also perceived favourably, as a comprehensive, ‘in‐depth’ focus on the lungs, ‘almost like a lung MOT’ (M_60_CS), although some misunderstood the detecting of other conditions incidentally as another goal of screening.

Participants also perceived the process as supportive due to the type of staff involved and the assumed roles they would play (e.g., nurses' informality yet expertize). This increased participants' trust, which some found motivating. The involvement of the GP similarly reassured participants, although due to the incorrect assumption that they mediate the entire LHC and LDCT process and results. This led some to intellectually outsource their decision‐making about the tests to their GP as their ‘primary source of support’ (M_57_CS).

**3. Anticipatory anxiety about screening results and further investigations**

3.6

Information describing abnormal screening results, further tests and associated risks, most frequently provoked anxious responses. Some participants found the description of abnormal results to be ‘blunt’ and ‘brutal’, and began to imagine themselves receiving these ‘frightening’ results and going to the hospital. Although some felt this information was important ('*they should tell people'* M_71_CS), others did not ‘think that's good information to give people’ (M_60_CS) and emphasized the need for this to be explained in person by a healthcare professional. Concern was exacerbated among those who found the results information hardest to understand, which in some cases promoted a fatalistic attitude towards the results being ‘like a lottery’. Additionally, some were concerned about the time it would take to receive results, during which ‘your nerves would be wrecked’ (M_56_FS), especially if further tests or surveillance were needed, accumulating into an unacceptably long period of uncertainty, which put a minority off attending. This was exacerbated by the term ‘shadows’, which implied it was unsafe to wait.

**4. Images intended to support comprehension provoked negative emotions**

3.7

Imagery can support comprehension and sustain attention but the imagery within this leaflet had a mixed reception. Photographic images of the CT scanner helped participants imagine what it would be like, reducing procedural anxiety. Other participants found this image claustrophobic and ‘overwhelming’ (F_55_FS), misinterpreting the scanner as an enclosed tunnel, which activated feelings of resistance to screening.

Biomedical images (e.g., lung diagram) sometimes aided comprehension (e.g., of the benefits of early diagnosis targeting only one lobe of the lung), but others described these as ‘too technical’ and irrelevant. A minority misinterpreted the lung diagram as showing cancer, an expectation that appeared to stem from prior exposure to damaged lung images used in smoking cessation campaigns, which provoked an anxious response.

Metaphorical images (e.g., images with question marks) frequently provoked confusion among participants who did not understand their significance. However, several participants interpreted metaphorical images positively (e.g., signifying different directions of decision‐making).

**5. Fatigue with smoking cessation information**

3.8

The inclusion of smoking cessation information did not adversely affect participants' responses to the leaflet or screening offer. However, there was a sense of fatigue from repeated exposure to similar messages, which meant this information failed to motivate participants to consider quitting. Although participants recognized cessation as important, many indicated that they did not intend to engage with services due to previous unsuccessful quit attempts or high dependence.

### 3.9 Engagement with the leaflet

Participants' attention was quickly drawn to the NHS branding, information about lung cancer prevalence and positive benefits of screening, consistent with System 1 type heuristics that the NHS can be trusted and screening is a good thing (see Table 4). Participants were subjectively observed as taking longer to deliberate about information regarding early diagnosis, less familiar symptoms of lung cancer and risks of screening that were previously unknown, which challenged their preconceptions. In doing so, this type of information engaged participants in slower, analytical System 2 type thinking for those able to understand the information as well as System 2 type emotional responses (see previous) among those overwhelmed by the information.

**1. Enough information to engage attention, support autonomous consideration and initiate shared decision‐making**

3.10

The leaflet was immediately perceived as trustworthy due to the NHS logo, which drew attention and motivated engagement ('*first thing I noticed it says National Health Service… the trust would be there, so I would be opening it'*, F_58_FS). Most participants found there to be ‘enough information to get you started… to go and have your lungs checked’ (M_71_FS), without it being overwhelming. As a result, most participants tentatively (e.g., '*I think'*) intended to attend, seeing the leaflet as positively influencing their decision, but expecting further information.

For most, the leaflet positively supported their autonomy and decision‐making without pressure, with participants valuing language that emphasized individual choice. The leaflet was also perceived to be useful for sharing decision‐making with family and friends, with some anticipating they would use the leaflet to approach their GP for support with the decision.

**2. Attentional bias towards incidence and early detection messages**

3.11

For most participants, lung cancer being common was new information that engaged attention, and motivated individuals to ‘take it [LHC offer] more serious’ (M_70_FS). The messages and statistics for early diagnosis also drew participants' focus, prompting deliberative thinking, as did the descriptions of treatment as more successful. Both were reassuring and motivated intentions to attend.

**3. Known risks downplayed, but unfamiliar harms prompted deliberative thinking and concern about screening reliability**

3.12

The importance of harm was often outweighed by ideologies, such as preferring to ‘be safe not sorry’, particularly for harms that felt familiar (e.g., radiation), with some dismissing their possibility completely: ‘why would there be cons?’ (F_55_CS). This appeared to partly stem from participants' implicit trust in the ‘system’, assuming the benefits must outweigh risks for the procedure to be offered and a bias towards medical intervention ('*better to be over cautious than not cautious enough'*, F_55_CS). Similarly, some participants perceived the risk of further unnecessary tests to be justified in ‘making sure’, leading a minority to perceive false positives and overdiagnosis relatively positively.

Unfamiliar harms tended to challenge this assumed benefit, with a renewed perspective that ‘the negatives would outweigh the positives’ (M_56_CS). For example, false negatives were deemed ‘scary’ yet ‘important’. Overdiagnosis was a particularly surprising concept, provoking anxiety and information avoidance for some, and conflicting with the assumption that cancer always causes harm. The level of worry depended on the frequency of overdiagnosis, which was not clear, and therefore undermined the perceived accuracy of lung cancer screening, ‘they can't even get the testing right, what's the point?’ (M_56_CS). Similarly, a few participants interpreted the number needed to screen (1 in 250) as signalling screening to be unreliable with a low perceived chance of benefit. Consequently, a minority were deterred from screening and suggested that complex and concerning harms are better communicated in person by a healthcare professional.

**4. Engagement in symptom appraisal and awareness**

3.13

The list of lung cancer symptoms engaged some participants in personal symptom appraisal. Some thought deliberatively about how lung cancer causes nonrespiratory symptoms, which opposed their understanding of how lung cancer affects the body. Some participants also questioned when they should present to their GP and with ‘one of them [symptoms] or all of them?’ (M_71_CS). Furthermore, many were unaware that there are no early symptoms of lung cancer, which engaged their attention.

### 3.14 Comprehension of images and information

Participants understood the roll out of the LHC and screening programme, and the main chronological steps including the different results of the LHC (see Table 5). Participants were confused by the rationale for, and basis of, eligibility for the LHC and lung cancer screening, as well as the meaning of a false negative. This partly stemmed from System 1 type assumptions about scans only being for those with symptoms and that imaging cannot miss cancer. Radiation risks, abnormal lung cancer screening results and frequencies of different screening outcomes were hard for participants to understand. Some participants engaged in deliberative System 2 type thinking supported by the presentation of frequencies, whereas others relied on prior knowledge of test results and harms or felt overwhelmed leading to their being guided by emotional responses.

**1. Understood the principle of an LHC and lung cancer screening**

3.15

Most participants understood that the LHC offering lung cancer screening was a new service beneficial for those with a smoking history. The ‘good little book’ clearly explained the procedural steps, letting ‘you know what actually happens and when’ (F_70_CS). This was supported by the chronological step‐by‐step order and formatting used to break down the information (e.g., numbering), emphasize key points and provide the gist (e.g., bold/colour emphasis). Participants also found the description of biennial screening reassuring, with some assuming screening would be ‘ongoing for the rest of your life’ (F_56_CS).

**2. Understood the types of LHC results but confused it with a symptomatic pathway**

3.16

Participants found the different LHC results ‘basic and understandable’ (M‐61_CS). However, some questioned the timeframe for receiving results and any referrals. While most understood that an offer of LDCT screening was not ‘guaranteed’ (F_58_CS), the rationale and criteria for eligibility were less clear, especially juxtaposed against information describing early diagnosis as beneficial. The LHC was often misinterpreted as a process designed to determine whether an individual has symptoms that clinically indicate screening. A minority of participants assumed that they would not need medical tests, including the LHC unless they had symptoms, with one participant insisting the leaflet clarify asymptomatic people are ineligible. However, some participants did understand the concept of screening for asymptomatic disease through experience with established cancer screening programmes.

**3. Misunderstood false negatives to be interval cancers**

3.17

The concept of a false‐negative result was new and surprising. While most understood the term, some found it difficult to believe a scan could miss cancer, particularly with no supporting explanation about ‘why it could get missed’ (F_59_FS), believing it more likely that the cancer is not present during the scan. The positioning of information about interval cancers directly after the description of false‐negative findings appeared to contribute to this misunderstanding, causing concern among those who conflated false negatives with interval cancers caused immediately by screening radiation.

**4. Poor understanding of radiation exposure due to an unfamiliar comparison**

3.18

The amount of radiation exposure from LDCT screening was poorly understood, which polarized participants' responses to this information. On one hand, the comparison to one year's exposure to the natural environment was reassuring and inferred to be ‘so minimal that it's worth it’ (F_59_FS). On the other, it generated concern about a large, concentrated exposure. In both cases, this comparison did not support comprehension. One participant suggested X‐rays as a more familiar and informative comparison. Indeed, some mistakenly assumed that the radiation exposure from an LDCT scan is equivalent to an X‐ray, inferring any harm as trivial. Many participants drew on prior knowledge of imaging tests and cancer treatment to understand the level of risk posed, with some assuming the radiation risk described to be from treatment if diagnosed, rather than screening.

**5. Conflating understanding of the different types of abnormal lung cancer screening results**

3.19

Participants sometimes mixed up the different types of abnormal results, both with each other and with the harms of screening due to their perceived similarities. For example, some questioned the difference between an abnormal result immediately suspicious for lung cancer and a pulmonary nodule requiring surveillance through repeat scans, conflating the two as concerning findings needing swift diagnostic tests.

Although the concept of a false‐positive result was generally understood, some misinterpreted it to mean another condition had been detected, conflating the term with incidental findings. Consequently, both were perceived as ‘beneficial’ (F_57_CS) outcomes. However, some found these findings alarming and sought an explanation as to the types of conditions found and how often.

**6. Outcome probabilities engaged deliberative thinking but overwhelmed those who found them too complex**

3.20

Participants varied in their ability to understand the icon array and accompanying numbers and text presenting the frequency of screening outcomes. Generally, the icon array facilitated understanding by visually illustrating the proportions. However, the inconsistency in reference groups used by accompanying natural frequencies, meant some found the information too ‘complicated’ and ‘like an exam paper’ (M_57_FS). The reference group of ‘250 people who have two low dose CT scans’ was particularly challenging to interpret and the basis of two scans more confusing than a single screening episode. Similarly, numbers that were not contextualized held little meaning.

## 4 DISCUSSION

This is the first study to explore engagement with, comprehension of, and responses to, NHSE's TLHC leaflet as standalone written information designed to support the knowledge component of informed choice about lung cancer screening. The principle and processes of lung cancer screening were well‐understood, with the leaflet prompting deliberative thinking, particularly in response to new information. However, there was also evidence that the leaflet did not promote a comprehensive understanding of screening. Participants tended to focus more on the benefits of screening and their comprehension of screening eligibility, harms and abnormal results was sometimes undermined by negative emotional reactions and cognitive heuristics (such as pre‐existing ideologies, experiences and assumptions about screening).

While participants valued the leaflet's emphasis on autonomy and described cautious intentions to engage in lung cancer screening, further information and discussion were expected with healthcare professionals. The need for interpersonal communication regarding the potential abnormal results and harms was emphasized, due to the anticipatory anxiety they evoked and difficulty interpreting this complex, fear‐inducing information alone. There was evidence of this information (and screening itself) being avoided, similar to research in colorectal cancer screening.^16^, ^20^, ^21^ This is problematic when the potential risks and outcomes must be communicated to support informed choice. When accompanying the screening invitation, the leaflet is also tasked with engaging a high‐risk population with a screening offer for feared cancer.^8^, ^9^ Relying on a single resource for both purposes is a difficult balance, especially when information is complex, new and emotionally charged. While this study only evaluated one example of a written information leaflet, the findings begin to suggest that a standalone written information leaflet may be important, but not sufficient, in supporting the comprehension of the screening offer. Future research could seek to further understand the sufficiency of standalone information and develop resources to support informed decision‐making as a relational process, rather than an individualistic discrete event.

The harms of screening (especially overdiagnosis), the asymptomatic basis for eligibility and incidental and indeterminate results were least understood. For some, this information challenged preconceptions of screening and overcame positive bias, engaging deliberative thinking. However, the different harms and results were often conflated, with pre‐existing assumptions biasing their interpretation and leading to their dismissal. For example, false‐negative results were misconstrued to mean interval cancers, and false‐positive results were perceived positively as incidental findings. A systematic review of studies of decision support tools,^11^ largely developed and tested in the US context, found issues with individuals' comprehension of similar aspects of screening, even though overall knowledge appeared improved. For example, in one study,^22^ 77% of participants misunderstood the eligibility criteria for screening after using the tool despite a substantial improvement in knowledge score. Crucially, our findings suggest that these difficulties in comprehension had consequences beyond how well‐informed screening participants might be, including negative emotional reactions, distrust, low confidence in screening reliability and discounting one's eligibility if asymptomatic. Indeed, when faced with ambiguous information an individual may be more likely to make an emotional decision;^10^, ^14^ with evidence this promotes suspicion and avoidance among those with lower numeracy.^23^, ^24^

Risk information in the form of numerical probabilities of screening outcomes (i.e., results or harms) was important in supporting deliberative thinking. These probabilities mostly reassured participants while sometimes appropriately deterring them if they found the frequency of harm unacceptable. The visual icon array facilitated understanding of proportions, but a complex reference group and the use of different denominators undermined comprehension and exacerbated negative emotional reactions. This is consistent with a best practice review recommending small, consistent denominators.^10^ However, when the absolute number is very small this can be challenging in practice. Using verbal evaluative labels can help improve comprehension when the precise probabilities are unknown or difficult to express, but the evidence for this is mixed.^10^, ^25^ Indeed, studies have shown that some participants still report confusion despite recommended numerical presentation styles for probabilities. Together, these findings underscore the need to pilot test materials with a diverse, representative population to ensure presentation techniques are applied in ways that suit the target audience.

The concurrent think‐aloud method and Dual Process Theory of cognition framework enabled in‐depth exploration of the emotional and cognitive drivers underlying participants' responses, among a diverse ‘screening naïve’ sample. The findings align with those from previous research showing that people appear to balance emotional responses with deliberative thinking when thinking about cancer.^26^ However, the hypothetical nature of the study means participants' responses may differ from those they would experience when actually invited to screening. The hypothetical invitation also meant that we were unable to measure informed choice among participants, which limits our understanding of its effectiveness in the real world. Furthermore, while we subjectively observed and interpreted potential cognitive and emotional biases guiding participants' responses, many occur outside of conscious awareness and could not have been articulated. Finally, we did not employ any comparative methods of data analysis, hence were unable to infer any variation in responses by participant characteristics, such as literacy or numeracy.

## 5 CONCLUSION

The single written NHSE TLHC information leaflet was found to be broadly acceptable in explaining the principles and procedures of lung cancer screening when offered in the context of NHSE's TLHC programme. However, while information about the harms and outcomes of screening prompted deliberative thinking, there was evidence of attentional biases and pre‐existing assumptions, which undermined their comprehension, as well as negative emotional reactions, promoting information avoidance and distrust. These findings suggest that in isolation, offering the NHSE TLHC information leaflet at the time of invitation to lung cancer screening may be inadequate in supporting informed decision‐making within the NHSE TLHC programme, which may require other interactions, types of resources and interpersonal strategies. The suggested recommendations in Table 6 are based on these findings within the NHSE TLHC programme specifically, but could begin to help direct the content of lung cancer screening information leaflets more widely, as well as broader multi‐modal strategies for supporting informed choice as a distributed process.

**Table 6:** Recommendations for the content and use of written lung cancer screening information

*Content of information leaflets for lung cancer screening*	
Eligibility	Clarify that an individual need not have symptoms to be eligible and distinguish the transition between the LHC and LDCT screening.
Procedural information	Simple, stepwise, chronological presentation sets clear expectations for the process.
Harms	Explain the difference between false‐negative results and interval cancers, and their frequency.
	Explain the difference between false‐positive results and incidental findings, their frequency and provide examples of these types of findings.
	Support understanding of the amount of radiation exposure from an LDCT scan by comparing it to more than one type of source and include relatively more familiar sources, such as perhaps, an X‐ray, flight and background radiation.
	When defining overdiagnosis, explain that it is not always possible to know which cancers do not cause harm and include the frequency.
Results	Explicitly distinguish between the different types of abnormal results that require further tests (e.g., diagnostic work‐up vs. surveillance) using distinct terminology.
Outcome probabilities	Use a consistent denominator and simple reference group (ideally a single screen).
	Position outcome probability information next to descriptions of the respective outcome so that the frequency can be immediately understood.
Choice of imagery	Imagery should be tested as it can provoke adverse emotional responses.
	Use photographic/pictorial imagery to demonstrate procedural information. Avoid metaphorical images and those perceived by a lay audience as too technical.
Smoking cessation	Test and use innovative and engaging smoking cessation messages for long‐term smokers, likely to have higher tobacco dependence.
*Using information leaflets for lung cancer screening*	
Interpersonal decision support	Include an avenue for, and assurance of, the opportunity to speak to a healthcare professional about lung cancer screening.
Multi‐modal and stage process	A written information leaflet should not be used in isolation to achieve an informed choice.
	If provided alongside the invitation to screening, the information leaflet's impact on uptake should be balanced with information exchange.
	Use different modes and formats to provide information that accommodates individual preferences for detail and type of information.

Abbreviation: LDCT, low‐dose computed tomography.

## AUTHOR CONTRIBUTIONS

Mbasan Jallow: Formal analysis, investigation, data curation, writing – original draft, project administration. Georgia Black and Sandra van Os: Conceptualization, methodology, formal analysis, investigation, writing – original draft, funding acquisition. Clara Kurtidu: Methodology, writing – review and editing. Kate E. Brain, David R. Baldwin, Michael Donnelly, Grace McCutchan, Kathryn A. Robband Samuel M. Janes: Conceptualization, methodology, writing – review and editing, funding acquisition. Samantha L. Quaife: Conceptualization, methodology, formal analysis, investigation, writing – original draft, supervision, funding acquisition.

## CONFLICTS OF INTEREST

Mbasan Jallow, Georgia Black, Sandra van Os, Kate E. Brain, Michael Donnelly, Clara Kurtidu, Grace McCutchan, Kathryn A. Robb and Samantha L. Quaife declares no support from financial organizations that might have an interest in the submitted work in the previous 3 years. Samuel M. Janes, is the Principal Investigator of an academic study (SUMMIT) which is sponsored and conducted by UCL and funded by GRAIL Inc., through a research grant awarded to Samuel M. Janes. Samantha L. Quaife collaborates on the SUMMIT Study and Mamta Ruparel has previously been supported as a researcher by funding for this study. Mamta Ruparel received travel funding for a conference from Takeda and an honorarium for planning and speaking at educational meetings from Astra Zeneca. Samuel M. Janes has been paid by Astra Zeneca, BARD1 Bioscience, Jansen and Achilles Therapeutics for being an Advisory Board Expert and travel to one US conference. Samuel M. Janes receives grant funding from Owlstone for a separate research study. David R. Baldwin has been paid by Astra Zeneca, BMS, MSD and Roche for educational events and advice. Georgia Black has been paid by Astra Zeneca for educational events. All authors perceive that these disclosures pose no academic conflict for this study. All authors declare no other relationships or activities that could appear to have influenced the submitted work.

## ETHICS STATEMENT

Ethics approval was provided by University College London (UCL) Research Ethics Committee (reference:17701/001). Participant's consent was also obtained.

## Supporting information

Supporting information.

Supporting information.

## Data Availability

Data that support the findings of this study are available on request from the corresponding author, and are not publicly available due to privacy or ethical restrictions.
