# Supplementary material for: Acceptability of a standalone written leaflet for the National Health Service for England Targeted Lung Health Check Programme: A concurrent, think‐aloud study
Source: Health Expect. 2022 Jun 2;25(4):1776–88. doi: 10.1111/hex.13520 (PMC9327842; doi:10.1111/hex.13520)
Supplement: Supplementary file 1 — Supporting information. [file HEX-25--s001.pdf]

What you need to know about your lung health check

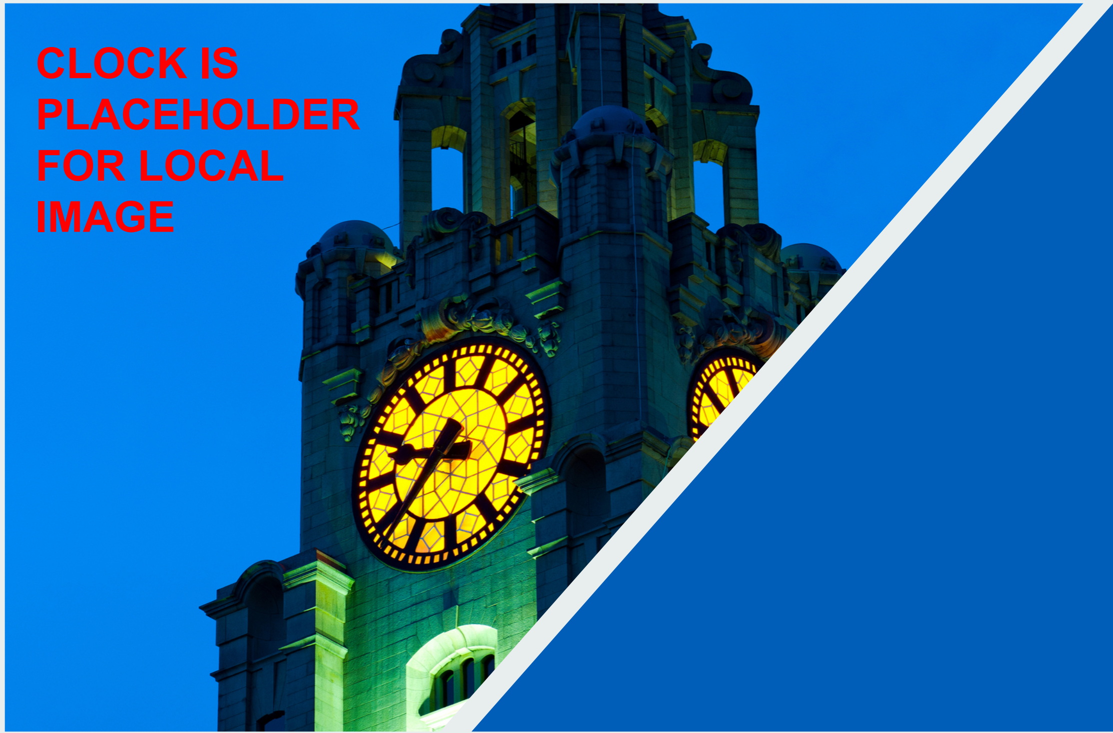

NHS England gateway reference: 000959

It is your decision whether to attend your lung health check or not.

This leaflet will help you decide.

Why are we offering lung health checks?

Run by specially-trained nurses, lung health checks aim to find out how well your lungs are working.

A lung health check can help find problems early – often before you notice anything is wrong.

If lung cancer, or another problem with your breathing or lungs is found early, treatment could be simpler and more successful.

Who do we invite?

Lung health checks are being offered in your area to people aged from 55 to less than 75 who smoke or used to smoke.

NHS lung health checks are different to NHS health checks.

Even if you've recently been for a health check, you should still consider attending your lung health check.

Space for local project information

What happens at a lung health check?

You will meet a nurse at your appointment. Your lung health check will take approximately 30 minutes.

1. The nurse will ask you some questions about your breathing and your overall lung health. They will ask you about your lifestyle, family and medical history.

2. They may look at how well you are breathing to find out whether there are any problems with your lungs. This is a simple test for which you blow into a hand-held machine called a spirometer. The machine measures how much air you breathe in and out.

3. The nurse may talk to you about having a lung scan. This checks for early signs of lung cancer and is called lung cancer screening.

4. They will ask you if we can keep your information on file to help us improve the lung health check programme.

You will have plenty of time to chat to the nurse and ask any questions.

You can bring a friend, family member or partner with you on the day if you want to.

Lung health check results

There are three possible results from your lung health check:

No problems found.
The nurse may find nothing to look into further. We will write to your GP so they know about the appointment.

Referral to your GP.
If problems with your breathing or lungs are found, the nurse may refer you on to your GP. We will write to your GP so they know about the appointment.

Offer of a lung cancer screening scan.
Whether or not you are offered a lung cancer screening scan will depend on your chance of getting lung cancer now or in the future. We work out your risk from your lifestyle, medical and family history. The nurse will help you to choose whether the test is right for you. We will write to your GP so they know about the appointment.

What is lung cancer?

Lung cancer is one of the most common types of cancer.

Lung cancer is when abnormal cells divide in an uncontrolled way to form a tumour in the lung.

There are usually no signs or symptoms in the early stages of lung cancer.

Finding cancer early means it is much more likely to be cured.

Most lung cancer grows slowly enough for it to be found at an early stage by a low-dose CT (Computed Tomography) scan.

Helping find lung cancer early is why we have set up this lung health check.

What is lung cancer screening?

Lung cancer screening uses a low-dose CT scan to check for the early signs of lung cancer, before you have any symptoms. This is a special scan that uses a very low dose of radiation and a computer to take a detailed picture of your lungs.

What happens during lung cancer screening?

1. During the scan, you will usually lie flat on your back on a bed that moves slowly into the CT scanner. The scanner is shaped like a ring and it rotates around a small section of your body as you pass through it.
2. Specially-trained staff (called radiographers) control the scanner from behind a screen in the CT room. You will be able to see and speak to them during the scan.
3. When a scan is taken, you will need to lie still and follow simple breathing instructions for 10 seconds. This makes sure the pictures are not blurred.
4. The scan is painless and you will be able to eat, drink or drive as normal after your scan.

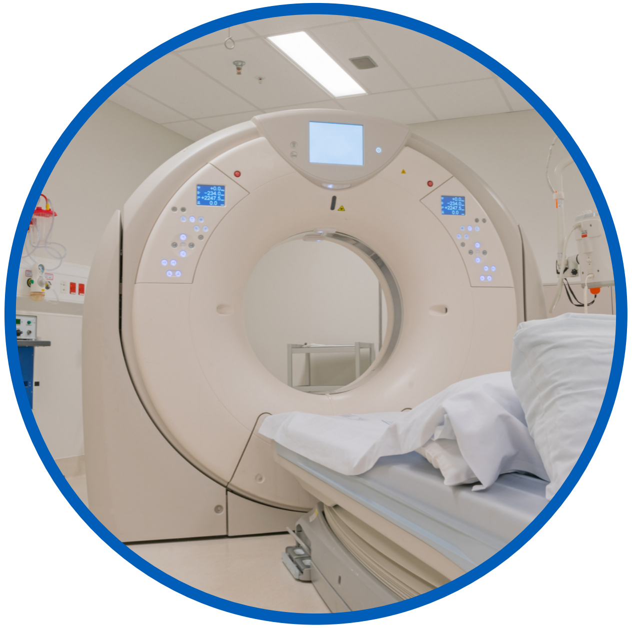

Lung cancer screening results

You will get your results within four weeks. There are four possible results:

No abnormalities found
This means that no signs of lung cancer or other abnormalities were seen on the scan. We will write to you with the results and ask you to come back for another scan in two years. However, it is still possible that lung cancer could develop or that the scan may have missed it. If you notice anything that is not normal for you before your next scan, tell your doctor.

Further scan needed
This usually means there are some shadows on the scan. It is probably something harmless but it could be more serious. We will write and ask you to come for another scan sooner than normal, in around three months, just to check.

Abnormal result
This means there is something abnormal on the scan and you need further tests. We will call you and write to the hospital so you can be seen by a doctor. It could mean you have lung cancer. Around half of people with an abnormal result will have lung cancer.

Incidental finding
This means there is no sign of lung cancer but there are signs of other problems on the scan that may need treatment or medical advice. We will write to you and your GP, you may need to see your GP or another doctor.

There are benefits and harms to lung cancer screening. You should read the information in this booklet to help you decide if you want to go for a screen if you are offered one.

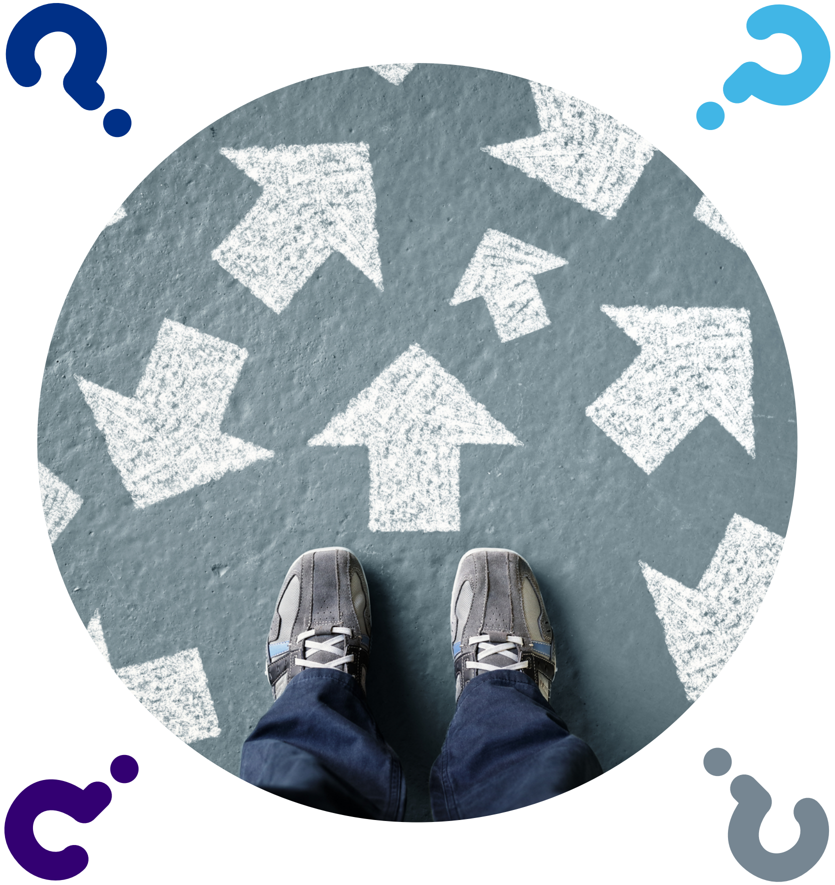

What are the possible benefits of lung cancer screening?

People are nearly three times more likely to be diagnosed with early stage lung cancer through lung cancer screening. Lung cancer found early can be cured.

- When found early, treatment may be simpler and more successful. Small and early lung cancer can often be removed completely by operation or treated with radiotherapy (using radiation to kill cancer cells) if found in time.
- Research suggests lung cancer screening reduces your risk of dying from lung cancer by around 25% after each scan.

Why early diagnosis makes a difference

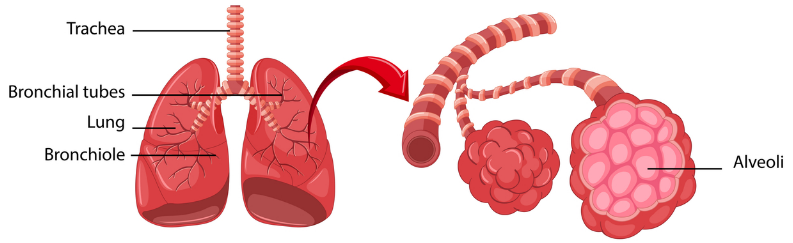

You have two lungs made of five sections called lobes.

Each lung is made up of thousands of grape-like sacs, called alveoli.

If there is a problem on one bit of the lung, early treatment can focus just on the bit that is affected.

The image above has been designed using resources from Freepik.com

What are the possible harms of lung cancer screening?

The low dose CT scan will expose you to a small amount of radiation. It is the same amount as about one year's worth of radiation from the natural environment.

Screening does not always find a cancer that is there and some people with lung cancer will be missed. This is called a false negative result and is very uncommon in lung cancer screening. Sometimes cancers can start to grow after screening.

In some people, the test picks up something even though they do not have lung cancer. This is called a false positive result and would mean you need further tests such as more scans or a biopsy. This can make people feel anxious or worried, and these further tests have risks.

As well as finding cancers that need treating, screening can also pick up lung cancers that will never cause a person harm. As a result, some people will be diagnosed and unnecessarily treated for lung cancer that would never otherwise have been found. This is called 'overdiagnosis'.

Helping you make a decision

What happens to 250 people who have lung cancer screening?

Research shows that for 250 people who have two low dose CT scans as they go through lung cancer screening:

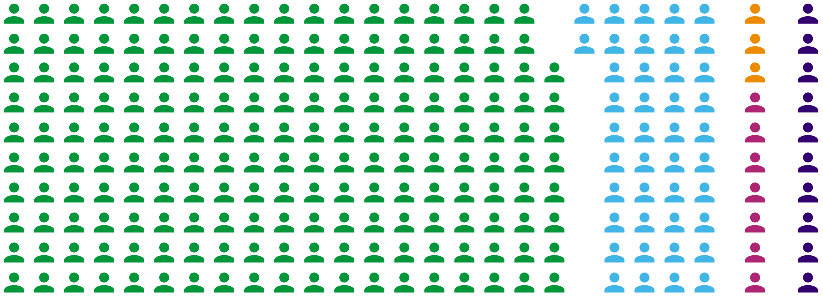

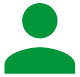

188 people will have no abnormalities at either scan.

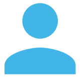

42 people will have an extra CT scan based on the results of the first one.

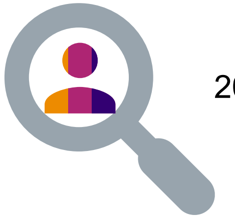

20 people will go to hospital for further tests.

Helping you make a decision

What will happen to the people that go to the hospital for further tests?

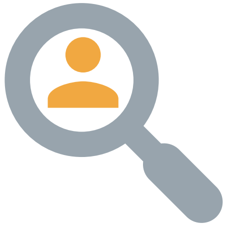

3 will have further scans but no tests. They will not have lung cancer.

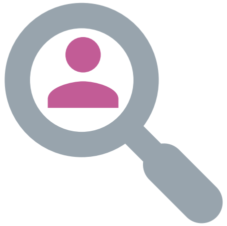

7 will have further tests such as a biopsy. They will not have lung cancer.

Less than 1 in 500 people will have an operation for suspected cancer but later be told that there was no cancer found.

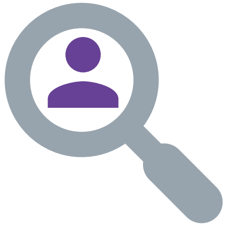

10 will have further tests such as a biopsy. They will have lung cancer. These people will be offered treatment - most often an operation - that can cure the cancer.

At least 1 more person for every 250 people screened will survive lung cancer if they had not been screened.

The NHS lung health checks will diagnose 3,400 lung cancers, many at an early stage.

What are the symptoms of lung cancer?

Lung cancer can start to develop between scans and screening can sometimes miss lung cancer. It is important to look out for anything that is unusual for you, especially:

- A persistent cough or change in your normal cough
- Coughing up blood
- Being short of breath
- Unexplained tiredness or weight loss
- An ache or pain when breathing or coughing
- Appetite loss

If you notice any of these changes, see your GP as soon as possible. Do not wait for another scan.

What can I do to reduce my risk of lung cancer?

The single best thing you can do to prevent lung cancer is not smoke.

If you do smoke and would like to stop, there is free, local and expert support available. The nurse at your lung health check can put you in touch with these services. You are three times more likely to quit smoking with help from a Stop Smoking Service.

Ask your GP about free and local support available.

Contact NHS Smokefree on 0300 123 1044 or visit www.nhs.uk/smokefree.

Want to know more?

For more information about lung cancer, lung health checks and advice on smoking visit:

NHS Lung Cancer Information
https://www.nhs.uk/conditions/lung-cancer/
NHS Smokefree
www.nhs.uk/smokefree
Cancer Research UK
www.cruk.org/lunghealthchecks
www.cruk.org/about-cancer/lung-cancer
www.cruk.org/smoking
Roy Castle Foundation
https://www.roycastle.org/help-and-support/lung-cancer-information/

Add local information on stop smoking services etc. here

Patient feedback recommends including a contact number

A privacy notice about this project can be found on the NHS England website https://www.england.nhs.uk/homepage/
