# Supplementary material for: Acceptability of a standalone written leaflet for the National Health Service for England Targeted Lung Health Check Programme: A concurrent, think‐aloud study
Source: Health Expect. 2022 Jun 2;25(4):1776–88. doi: 10.1111/hex.13520 (PMC9327842; doi:10.1111/hex.13520)
Supplement: Supplementary file 2 — Supporting information. [file HEX-25--s002.docx]

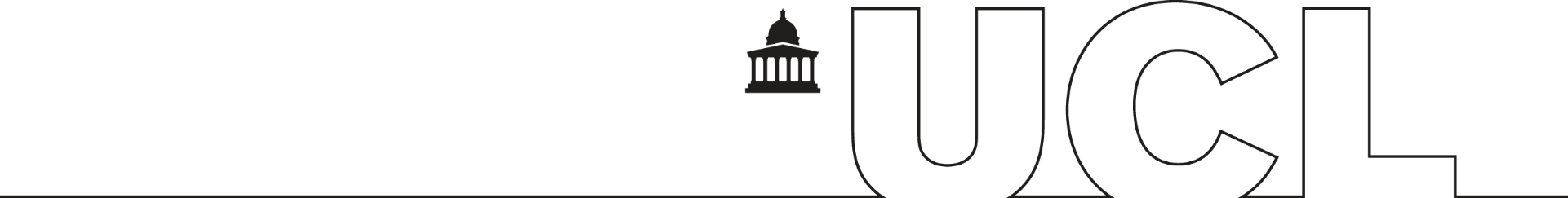


**DEPARTMENT OF BEHAVIOURAL SCIENCE AND HEALTH**

**Supplementary File 1: Think Aloud Interview Topic Guide**

**Evaluating a Lung Health Check information leaflet**

**This document is intended to be a guide. The following topics/questions/prompts are not exhaustive and the researcher may probe or follow the participants’ line of interest as is appropriate for the purpose of this study.**

1. **Introduction to study**

- Introduce self and briefly explain project.
- Provide all potential participants with a copy of participant information sheet
- Answer any questions and explain process.
- Remind participants of their rights, including confidentiality, right to withdraw, etc.

**After full explanation of project has been given, if participant says they will take part:**

- Ask participant to read and sign participant consent form.

1. **Pre-task interview**

*Health information preferences and needs*

- Including amount, format, mode, style

*Preferred role in decision-making about health*

- Including personal role, involvement of others (e.g., family/friends/HCPs), recommendations

1. **Introduction to ‘Think Aloud’ approach and practice task**

“*In a minute, I will give you an envelope containing a leaflet to read. I would like to emphasise that this is not a test; I am simply interested in getting your views on the materials. I would like you to read the materials while “thinking aloud”. This means that I would like you to look over the materials, as if you had just received them in the post, and while you do so, say what you are thinking and feeling. You may read the text out loud if you would like to, but there is no need to do so if you’d prefer not to. I may take some notes while you are thinking aloud. However, I would like you to imagine that you are at home and that I am not here. It’s important that you are honest when ‘thinking aloud’. There’s no need to be polite. You can even swear if that’s what you’re thinking. Your honesty is very valuable to us.*

*If you are silent for any long period of time, I will prompt you by asking: ‘what are you thinking now?’*

*Thinking aloud usually feels a bit strange at first, as it is an unusual task. Don’t worry about it, most people quickly get used to it. We will start off with a* ***practice task*** *to make sure that you feel comfortable. I would like you to look over one of these leaflets while trying to say what you are thinking and feeling*.”

1. **‘Think Aloud’ interview task**

*“Thank you. Now, I would like you to imagine that you are at home and have received this envelope in the post. To help with this, try to think about how you receive your post. For example, does it come through your front door, or does it go to a post-box for collection? With this in mind, please open the envelope and look through the contents while thinking aloud.”*

*Prompt/probe thoughts/feelings/interpretation/comprehension*

- Example probes and prompts:
- I noticed that you mentioned that you thought that [...]Can you tell me a bit more about that?
- I noticed that you made a comment about [...]. Can you elaborate on that?

1. **Post-task interview**

*Comprehension*

- Do you have any initial thoughts/comments about the leaflet?
- Did you find any part of the leaflet difficult to understand?
- **[**IF YES] Did you find the leaflet difficult to understand as a whole, or particular sections or words that were used?
- [IF YES] Can you repeat that section in your own words?
- [IF YES] Can you think of a better way of giving that information?
- Do you think the information is clear or would you prefer this to be changed (and how/why)?
- [IF YES] Can you think of a better way of phrasing/showing the information?
- What do you think is meant by [XXX]?
- Can you think of another word for [XXX]?

*Outstanding information needs/areas for clarification*

- Do you have any questions about Lung Health Checks/lung cancer screening?
- What, if anything, is missing from the leaflet?
- To what extent do you feel able to make a decision about attending?

*Future Lung Health Check intentions*

- What do you think you would do next after reading the leaflet? Look for further information?
- Do you think that you would attend a Lung Health Check if you were invited? Why/why not?
- If unsure: What is mainly stopping you from saying yes or no?

*Preferences/alternative formats of information materials*

- Which of these leaflets/formats/styles do you prefer? Why?
- Can you think of a better way to provide that information?

*Views about Lung Health Check data being used for research purposes*

- The NHS would like to make anonymous information collected from patients in the Lung Health Check programme to be available for research purposes, how do you feel about this? Why?

1. **Close and debrief**

- Thank you
- Explain what will be done with information
- Remind where contact details can be found
- Check participant is happy and answer any questions.
